# Maize AKINβγ Proteins Interact with P8 of Rice Black Streaked Dwarf Virus and Inhibit Viral Infection

**DOI:** 10.3390/v12121387

**Published:** 2020-12-04

**Authors:** Mingjun Li, Xi Sun, Dianping Di, Aihong Zhang, Ling Qing, Tao Zhou, Hongqin Miao, Zaifeng Fan

**Affiliations:** 1 State Key Laboratory of Agro-Biotechnology and Key Laboratory of Pest Monitoring and Green Management-MOA, China Agricultural University, Beijing 100193, China; sunxi1994@126.com (X.S.); taozhoucau@cau.edu.cn (T.Z.); 2 Chongqing Key Laboratory of Plant Disease Biology, College of Plant Protection, Southwest University, Chongqing 400716, China; qling@swu.edu.cn; 3 Plant Protection Institute, Hebei Academy of Agricultural and Forestry Sciences, Baoding 071000, China; chmrdy@yahoo.com.cn (D.D.); zhangaihong08@163.com (A.Z.); miao78596@yahoo.com.cn (H.M.)

**Keywords:** rice black streaked dwarf virus, P8 protein, ZmAKINβγ, protein interaction, viral accumulation, primary carbohydrate metabolism

## Abstract

Rice black streaked dwarf virus (RBSDV) is an important agent causing maize rough dwarf disease, whereas the host factors responding to RBSDV infection are poorly understood. To uncover the molecular interactions between RBSDV and maize, a yeast two-hybrid screen of a maize cDNA library was carried out using the viral P8 protein as a bait. ZmAKINβγ-1 and *ZmAKINβγ-2* (βγ subunit of *Arabidopsis* SNF1 kinase homolog in maize) possessing high sequence similarities (encoded by two gene copies) were identified as interaction partners. Their interactions with P8 were confirmed in both *Nicotiana benthamiana* cells and maize protoplasts by bimolecular fluorescence complementation assay. The accumulation levels of *ZmAKINβγ* mRNAs were upregulated at the stage of the viral symptoms beginning to appear and then downregulated. ZmAKINβγs are putative regulatory subunits of the SnRK1 complex, a core regulator for energy homeostasis. Knockdown of *ZmAKINβγ*s in maize regulated the expression levels of the genes involved in sugar synthesis or degradation, and also the contents of both glucose and sucrose. Importantly, downregulation of *ZmAKINβγ*s expressions facilitated the accumulation of RBSDV in maize. These results implicate a role of *ZmAKINβγ*s in the regulation of primary carbohydrate metabolism, and in the defense against RBSDV infection.

## 1. Introduction

Maize (*Zea mays* L.) is the most widely grown crop worldwide and is one of the most important food, forage, source of biofuel and other industrial materials [1]. However, a variety of diseases pose serious threats to maize production and quality. Among them, maize rough dwarf disease (MRDD) is a widespread and destructive viral disease worldwide [2,3]. It has been reported that the causal agents of MRDD in China are two fijiviruses (in the family of *Reoviridae*) including rice black streaked dwarf virus (RBSDV) and southern rice black-streaked dwarf virus (SRBSDV) [4,5,6]. RBSDV is an important and devastating viral pathogen infecting maize, rice, wheat and some other graminaceous hosts, which can be propagatively transmitted by small brown planthopper (*Laodelphax striatellus*) [7,8]. Maize plants infected by RBSDV exhibit severe growth abnormalities, including plant dwarfing, dark green leaves, and waxy white tumors on sheaths and veins of the abaxial surface of the leaves [9,10]. RBSDV has a double-shelled, icosahedral capsid approximately 75 to 80 nm in diameter and contains 10 segments of linear double-stranded RNAs (dsRNAs) ranging from 1.8 to 4.5 kbp in length [5,11]. It has been reported that 13 proteins can be encoded by these genomic segments, and the knowledge on the functions of most of these proteins is limited to date. As previously reported, protein P8 encoded by genomic segment 8 is the minor core protein of RBSDV virion, whose content in purified virion was much less than that of P2, another component of the core protein [12,13]. Besides, P8 can localize to the nucleus of insect and plant cells as homodimers and repress transcription from GAL4 promoter in suspension cells of tobacco (Bright Yellow-2) [14]. In addition, a recent report showed that SP8 encoded by SRBSDV, a closely related fijivirus, can disturb the auxin pathway and facilitate virus infection by directly interfering with the dimerization of auxin response factor 17 of rice (OsARF17) [15]. However, the molecular interaction between RBSDV P8 and its host plants remains elusive.

The sucrose nonfermenting-1 protein kinase (SNF1) in yeast, AMPK (AMP-activated protein kinase) in mammals, and SnRK1 (SNF1-related protein kinase 1) in plants belong to a conserved protein kinase family, that can function as energy sensors and regulators for maintaining the cellular and organismal energy homeostasis in eukaryotes [16,17,18]. For this function, it has been shown that SNF1/AMPKα/SnRK1α kinase functions as catalytic subunit by forming a complex with two other subunits, typically the β and γ [19]. In plants, however, except for the structurally typical γ subunit, an atypical γ subunit, referred to as SnRKβγ or AKINβγ, was evolved to form SnRK1 complex [20]. AKINβγ protein comprises the conserved four CBS (cystathionine-β synthase) domain, which is characteristic of γ subunit and an extended N-terminal region with homology to the CBM (carbohydrate binding module) domain of the β subunit, which was regarded as a structural domain fusion of β and γ subunits [16,18,20,21]. Intriguingly, the *Arabidopsis thaliana* hybrid AKINβγ rather than the conventional AKINγ subunit was reported to function as the canonical γ subunit, which is involved in SnRK1 complex formation and SnRK1 signaling in *Arabidopsis* [21,22].

In previous reports, the crucial roles of SnRK1 kinase in biotic and abiotic stress response, seeds germination and seedling growth, and organ development, through direct interaction with some plant proteins or pathogenic factors, have been uncovered [23,24,25,26,27]. For the function of AKINβγ subunit in plants, in addition to its role in SnRK1 complex formation, some progresses on the characteristics of this protein has been achieved in *Arabidopsis* and maize. It has been reported that AtAKINβγ could localize to chloroplast of mesophyll cells and bind starch, and interestingly, starch binding could inhibit the SnRK1 catalytic activity [28]. In addition, AtAKINβγ could interact with two pathogen resistance proteins, AtHSPRO1 and AtHSPRO2, through its CBM domain, suggesting its role in plant pathogen resistance [21]. Two copies of the *AKINβγ* gene located in different chromosomes in maize, encoding ZmAKINβγ-1 and *ZmAKINβγ-2* proteins with high sequence homology [20]. These two proteins can complement the yeast mutant deficiency of snf4, a homologue of ZmAKINβγ, and can also interact with the catalytic subunit ZmSnRK1, suggesting their likely roles in SnRK1 complex formation and signaling [29].

In this study, we identified two members of ZmAKINβγ, ZmAKINβγ-1 and *ZmAKINβγ-2*, in maize, that interact with RBSDV P8 in yeast, *N. benthamiana* and maize protoplasts. The relative expression levels of *ZmAKINβγ*s in RBSDV-infected to mock-inoculated maize plants were examined by RT-qPCR. Further, virus-induced gene silencing (VIGS) mediated down-regulation of the *ZmAKINβγ*s expression levels were performed to determine the function of *ZmAKINβγ*s in primary carbohydrate metabolism regulation and the response to RBSDV infection. Our results contribute to further understanding of the biological functions of *ZmAKINβγ* genes and the possible relevance of primary carbohydrate metabolism to RBSDV infection.

## 2. Materials and Methods

### 2.1. Plasmid Construction

The open reading frame sequences of *ZmAKINβγ-1* (GenBank accession number: AF276085.1) and *ZmAKINβγ-2* (GenBank accession number: AF276086.1) in maize, and their homologous in rice (*OsAKINβγ*, GenBank accession number: XM_015775494.2) and *N. benthamiana* (*NbAKINβγ*, GenBank accession number: MT408913) were amplified, respectively. For Y2H assay, the coding sequence of RBSDV P8 (GenBank accession number: KC134296.1) was amplified and inserted into NcoⅠ-BamHⅠ site of pGBKT7 vector to produce DNA binding domain-fused ORF (open reading frame) construct. The coding sequences of ZmAKINβγs and the truncate mutants of *ZmAKINβγ-2*, including *ZmAKINβγ-2*(1-153), *ZmAKINβγ-2*(154-496), *ZmAKINβγ-2*(1-237), *ZmAKINβγ-2*(1-333), *ZmAKINβγ-2*(1-415), and *ZmAKINβγ-2*(1-454) were cloned into EcoRⅠ-BamHⅠ site of GAL4 active domain vector pGADT7. The ORF sequences of OsAKINβγ and NbAKINβγ were inserted into ClaⅠ-XhoⅠ site and ClaⅠ-BamHⅠ site of pGADT7 vector, respectively.

For BiFC assay, the coding sequences of P8 and ZmAKINβγ were inserted into the SpeⅠ-XhoⅠ site of pSPYNE and pSPYCE and were used in *N.benthamina*. For BiFC assay in maize protoplasts, another pair of vector sreferred to as pUC-SPYNE and pUC-SPYCE was used with the same cloning strategy.

To determine the subcellular localization of P8 and ZmAKINβγs in maize protoplasts and *N.benthamiana*, P8 was fused at the C terminus of GFP (green fluorescence protein) by respectively inserting the ORF of GFP and P8 into the BglⅡ-HindⅢ and PstⅠ-BamHⅠ site of pGD vector, and the coding sequences of ZmAKINβγs were also cloned into pGD vector to produce recombinant proteins with GFP or RFP at the C terminus.

For BMV VIGS assay, a fragment of highly conserved nucleic acid sequence between two *ZmAKINβγ* genes, with the size of 180 base pair (bp), was amplified from AD-*ZmAKINβγ-2* construct and inserted into the AvrⅡ-NcoⅠ site of BMV VIGS vector pC13/F3, to produce BMV-ZmAKINβγs [30,31].

The constructs and primers used in this study are listed in Supplemental Table S1. The primers used for RT-qPCR are provided in Supplemental Table S2.

### 2.2. Plant Growth and Virus Inoculation

Maize plants growth and virus inoculation were carried out as described previously with slight modifications [32,33]. Briefly, RBSDV-infected wheat plants were collected as a virus source from Hebei Province, China. Maize inbred line Zheng 58 were exposed to *L. striatellus* carrying RBSDV at two-leaf stage for 3 days in specific inoculation chambers. The inoculated plants were grown in a greenhouse (26 °C day and 22 °C night, 16-h-light/8-h-dark cycle) after removing the insects completely.

### 2.3. Yeast Two-Hybrid Assay

The maize cDNA library screening and the positive interactors identification was performed as instructed by the manufacturer (Matchmaker^®^ Gold Yeast Two-Hybrid System User Manual, Clontech, Mountain View, CA, USA). The interaction analyses of two proteins in yeast were implemented as described previously [34].

### 2.4. Confocal Microscopy

For BiFC and subcellular localization assays performed in *N. benthamiana* leaves and maize protoplasts, fluorescence signals were examined using an Olympus FluoView 1000 confocal microscope (Olympus, Tokyo, Japan) equipped with Olympus FluoView software FV10-ASW 4.0 Viewer. The GFP and RFP fluorescence were captured in the EGFP and RFP channels, respectively. Because of the weak expression of P8 in plant cells, the images of BiFC and the subcellular localization of P8 were captured at 96 h post agroinfiltration of the *N. benthamiana* leaves. For subcellular localization assays in *N. benthamiana*, the GFP and RFP fluorescence signals were excited at wavelength of 488 nm and 543 nm, respectively, with laser intensity of 47%. For the protein expression in maize protoplasts, the fluorescence signals were visualized at 20 h post transformation. All the images were processed using Adobe Photoshop software. At least two independent experiments were performed.

### 2.5. BMV-Induced Target Gene Silencing Coupled with RBSDV Inoculation in Maize

Brome mosaic virus (BMV)-mediated VIGS assay in maize was performed as previously reported [35]. Briefly, *Agrobacterium tumefaciens* cultures carrying BMV-ZmAKINβγs or BMV-GFP was co-infiltrated with pC13/F1+2 into *N. benthamiana* leaves. At 3 dpi, the infiltrated leaves were collected for BMV virion isolation and the subsequent rub-inoculating to maize seedlings (inbred line Va 35) at two-leaf stage. After preservation of moisture for 6 days in a chamber (20 °C day and 18 °C night, 16-h-light/8-h-dark cycle), these plants were subjected for RBSDV inoculation using viruliferous *L. striatellus*. RBSDV inoculation was conducted as presented above. At 2 weeks after RBSDV infection, the third true leaves of each plant were collected for further analyses.

### 2.6. Maize Protoplasts Preparation and Transformation

Maize protoplast preparation and transformation were performed as described previously [35,36].

### 2.7. RNA Extraction and RT-qPCR Analysis of Gene Expression

Total RNA was extracted from maize tissue using TRIzol reagent (Invitrogen, Foster City, CA, USA) and subsequently treated with RNase-free DNase I (Takara Bio, Mountain View, CA, USA) as instructed by the manufacturer. A total RNA of 2 μg was used to synthesize the first-strand cDNA with an oligo (dT) primer for host genes using M-MLV reverse transcriptase (Promega, Madison, WI, USA). For reverse transcription of RBSDV RNAs, random primesr were used because of the lack of a poly (A) tract at the viral genomic RNA’s 3′ end. RT-qPCR and data analyses were performed as previously reported [37]. The transcriptional level of *ZmUBI* (*ubiquitin*) was used as an internal control. The relative expression levels of target genes were calculated using the 2^−ΔΔCT^ method [38]. The experiments were replicated at least three times. The primers used for RT-qPCR analysis are listed in Supplemental Table S2.

### 2.8. Western Blot Analysis

The protein extraction from maize leaves and western blot assay were performed as described previously [39].

### 2.9. Analyses of Nonstructural Soluble Carbohydrates and Starch

The accumulations of nonstructural soluble carbohydrates including glucose, fructose, and sucrose in maize leaves were determined using the method as described previously [40,41].

For starch quantification, BMV- or RBSDV-inoculated maize plants were kept in darkness for 4 h before detection [42]. The Starch content was measured using a total starch assay kit (Megazyme, Berkshire, UK) as instructed by the manufacturer.

## 3. Results

### 3.1. RBSDV P8 Interacts with ZmAKINβγs

**Figure 1:**
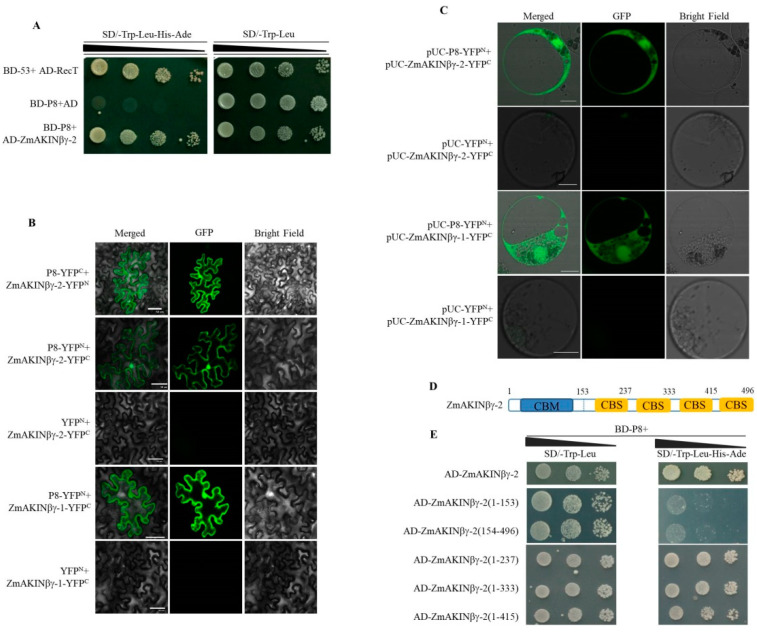
Rice black streaked dwarf virus (RBSDV) P8 interacts with ZmAKINβγ in yeast and in planta. (**A**) Interactions between *ZmAKINβγ-2* and P8 of RBSDV in yeast. The plasmid combinations indicated at the left side of the image panel were co-transformed into yeast strain Y2H Gold, and the transformants were spotted on the selective medium SD/-Trp/-Leu and SD/-Trp/-Leu/-His/-Ade with 10-fold serial dilutions and grown at 30 °C for 3–4 d. (**B**) Bimolecular fluorescence complementation (BiFC) of interaction between RBSDV P8 and *ZmAKINβγ-1* or *ZmAKINβγ-2* in *N. benthamiana*. Different BiFC vector combinations were co-agoinfiltrated into *N. benthamiana* leaves, and YFPfluorescence signal was captured at 96 h post infiltration. Bars = 50 μm. (**C**) BiFC of interaction between RBSDV P8 and *ZmAKINβγ-1* or *ZmAKINβγ-2* in maize protoplasts. Different BiFC vector combinations as indicated were co-tranfected into maize protoplasts and the fluorescence signal was captured at 20 h post tranfection. Bars = 10 μm. (**D**) Schematic representation of the functional motifs in *ZmAKINβγ-2* and of the truncated mutants. The numbers represent the positions of amino acids in full length coding sequence of *ZmAKINβγ-2*. (**E**) Interactions between P8 and *ZmAKINβγ-2* truncated mutants in yeast. The plasmid combinations indicated at the left side of the image panel were co-transformed into yeast strain Y2H Gold, and the transformants were spotted on the selective medium SD/-Trp/-Leu and SD/-Trp/-Leu/-His/-Ade with 10-fold serial dilutions and grown at 30 °C for 3–4 d.

To identify the host proteins interacting with RBSDV P8, a yeast two-hybrid screen of a maize cDNA library was performed using the P8 protein fused to the GAL4 DNA binding domain (BD-P8) as bait. One clone with sequence aligned to maize AKINβγ-2 was identified. To confirm this result, the coding sequence of *ZmAKINβγ-2* was cloned from maize and fused to the GAL4 activation domain (AD-*ZmAKINβγ-2*), and the interaction between P8 and *ZmAKINβγ-2* was verified by the subsequent yeast two-hybrid assay (Y2H, Figure 1A).

To evaluate the interaction between P8 and *ZmAKINβγ-2* *in planta*, bimolecular fluorescence complementation (BiFC) assays were performed in *N. benthamiana* leaves. P8 and *ZmAKINβγ-2* were fused to N terminal fragment of yellow fluorescence protein (YFPN) and YFPC, to generate P8-YFPN, P8-YFPC, *ZmAKINβγ-2*-YFPC, and *ZmAKINβγ-2*-YFPN. As a result of *Agrobacterium*-mediated infiltration coupled with observation using laser scanning confocal microscope, the YFP fluorescence could be observed in the nucleus and cytoplasm of P8-YFPN and *ZmAKINβγ-2*-YFPC or P8-YFPC and *ZmAKINβγ-2*-YFPN co-infiltrated *N. benthamiana* leaf epidermal cells, at 4 days post infiltration (dpi) (Figure 1B). In contrast, no fluorescence signal was detected in negative controls, that YFPN and *ZmAKINβγ-2*-YFPC co-infiltrated *N. benthamiana* leaf cells. The BiFC assay was further performed in maize protoplasts, to verify the interaction between P8 and *ZmAKINβγ-2* in the natural host of RBSDV. The coding sequences of P8 and *ZmAKINβγ-2* were introduced into another set of split YFP vectors, pUC-SPYNE and pUC-SPYCE, respectively, to produce pUC-P8-YFPN and pUC-*ZmAKINβγ-2*-YFPC, respectively. In accordance with the BiFC results shown in *N. benthamiana* leaf cells, the YFP fluorescence could be observed in the nucleus and cytoplasm of pUC-P8-YFPN and pUC-*ZmAKINβγ-2*-YFPC co-transfected maize protoplast cells at 20 h post transfection (hpt). No YFP fluorescence signal could be observed in pUC-YFPN and pUC-*ZmAKINβγ-2*-YFPC transfected maize protoplasts, which served as negative control (Figure 1C).

It has been reported that two AKINβγ proteins, named ZmAKINβγ-1 and *ZmAKINβγ-2*, with high sequence identity were encoded by maize [20]. We cloned the open reading frame (ORF) sequence of *ZmAKINβγ-1* mRNA and confirmed the interaction between ZmAKINβγ-1 and P8 in planta. As revealed by the observation of YFP fluorescence in the nucleus and cytoplasm of P8 and ZmAKINβγ-1 co-expressed plant cells, the in vivo interaction between these two proteins was also confirmed in BiFC assays implemented in *N. benthamiana* leaf cells and maize protoplasts (Figure 1B,C).

It has been known that AKINβγ is characterized by two types of motifs, conserved CBM and CBS motifs. Motif analysis (https://www.ebi.ac.uk/interpro/) revealed that four CBS domains and one N-terminal CBM are involved in *ZmAKINβγ-2* protein (Figure 1D), and Y2H assay was performed to determine the domain in *ZmAKINβγ-2*, which is responsible for the interaction with P8. The result showed that the truncated protein including CBM and at least the first one CBS domain is necessary to interact with P8 (Figure 1E).

AKINβγ proteins are conserved γ subunits across the plant kingdom. To explore the interaction specificity between RBSDV P8 and AKINβγ proteins, the homologues of ZmAKINβγ from rice, another natural host of RBSDV, and from *N. benthamiana,* which is a non-host of RBSDV, were cloned. The amino acid sequence multiple alignment of AKINβγ proteins encoded by maize, rice (designated as OsAKINβγ), and *N. benthaminana* (designated as NbAKINβγ) revealed high sequence similarity in the CBM and CBS domain (Figure S1). Positive interactions between P8 with both tested AKINβγs occurred in yeast, suggesting the interactions between RBSDV P8 and AKINβγ proteins are not host-plant specific (Figure S2A). Y2H assay was also used to determine the interaction between *ZmAKINβγ-2* and some other viral proteins, except for P8, encoded by RBSDV S5 to S10, and no interaction was observed (Figure S3). In addition, we cloned the ORF sequence of SRBSDV S8 encoding SP8 protein, which has 71.24 % amino acid sequence homology with RBSDV P8, and confirmed its interaction with ZmAKINβγ-1 and -2 (Figure S2B).

### 3.2. RBSDV Infection Regulated the Expression of ZmAKINβγ Genes

**Figure 2:**
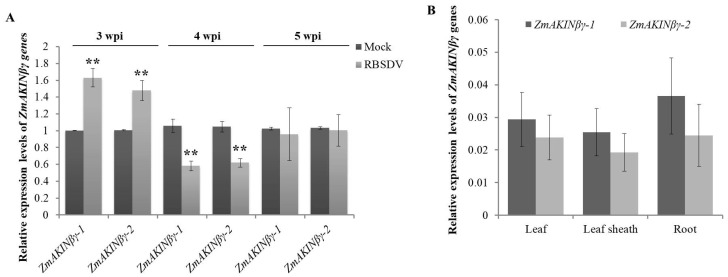
The relative expression levels of **ZmAKINβγ-1** and *-2* in RBSDV-infected maize plants at different growth stages. (**A**) and in different maize organs (**B**). (**A**) Relative expression levels of **ZmAKINβγ-1** and *-2* in RBSDV-and mock-inoculated maize plants were determined by RT-qPCR at three, four, and five weeks post inoculation (wpi). (**B**) The expression of **ZmAKINβγ-1** and *-2* in different organs of two-week-old maize cv Va35. The expression levels of two *ZmAKINβγ* genes were determined by qRT-PCR and the relative transcription levels were normalized using maize *ubiquitin* (*ZmUBI*) mRNA as a reference. Three independent experiments were conducted with at least three biological replicates for each treatment. Error bars represented the means ± SE. Significant differences were indicated using Student’s *t*-test: ** indicates *p* < 0.01. UBI was used as the internal reference.

To explore the response of *ZmAKINβγ* to RBSDV infection, the relative transcription levels of two *ZmAKINβγ* genes in RBSDV-infected to mock-inoculated maize plants were determined. For this purpose, we inoculated maize seedlings at two-leaf stage with RBSDV-infected or RBSDV-free *L. striatellus* insects for three days, respectively, and the viral symptom appeared as white wax swelling on the back of RBSDV-infected leaves could be observed at about 20 days after the insects were removed (designated as 20 dpi, Figure S4). The third true leaves were collected at three, four, and five weeks post inoculation (wpi) from RBSDV- and mock-inoculated plants and subjected to reverse transcription-quantitative real-time PCR (RT-qPCR) using the primer pairs specific to *ZmAKINβγ-1* and *ZmAKINβγ-2*, respectively. In our results, parallel regulation models for these two *ZmAKINβγ* genes responding to RBSDV infection were revealed. The *ZmAKINβγ* genes were upregulated at 3 wpi, and were downregulated at 4 wpi, suggesting the distinct roles of ZmAKINβγ in plant response to RBSDV infection at different infection stages (Figure 2A).

In order to investigate whether ZmAKINβγ could respond to infections by other viruses, two other phylogenetically unrelated maize-infecting viruses, SCMV (sugarcane mosaic virus), a potyvirus, and MCMV (maize chlorotic mottle virus), a member of the genus *Machlomovirus* (family *Tombusviridae*), were used to inoculate maize plants, respectively. Interestingly, the expression levels of two *ZmAKINβγ* genes could be significantly downregulated by SCMV at 7 and 10 dpi. During MCMV infection, the transcription levels of *ZmAKINβγ*s were upregulated at 9 dpi and were downregulated at 11 and 13 dpi (Figure S5). These results suggested that *ZmAKINβγ* might be involved in conserved physiological pathways such as cellular energy homeostasis, which can have broadspectrum responses to different biotic stresses.

Previous report showed a similar combined expression level of two *ZmAKINβγ* genes in different maize tissues [20]. In this work, the expression levels of *ZmAKINβγ-1* and *ZmAKINβγ-2* were determined by RT-qPCR, respectively, in different tissues (leaf, leaf sheath, and root). As a result, no significant tissue expression preference was observed for both *ZmAKINβγ* genes (Figure 2B and Figure S5).

### 3.3. The Subcellular Localization of ZmAKINβγ Proteins

**Figure 3:**
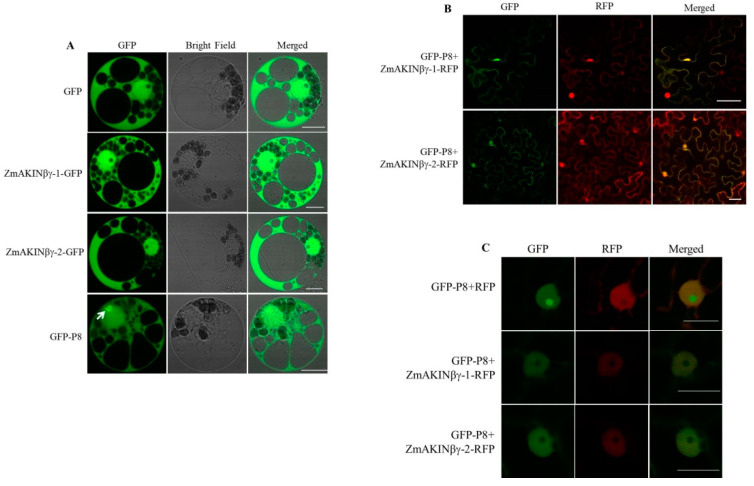
Subcellular localization of ZmAKINβγ and P8 in planta. (**A**) Subcellular localization of ZmAKINβγ and P8 in maize protoplasts. Maize protoplasts were transfected with vector combinations as indicated. GFP fluorescence signal was detected at 20 h post transfection. Bars = 10 μm. The white arrow indicates the nucleolus. (**B**,**C**) The co-localization of P8 and **ZmAKINβγ-1** or *-2* in epidermic cells (**B**) and the cell nucleus (**C**) of *N. benthamiana* leaves. GFP fluorescence signal was detected at 96 h post infiltration. Bars = 50 μm in B, and Bars = 20 μm in C.

In order to determine the subcellular localization of ZmAKINβγ-1 and *ZmAKINβγ-2* proteins in the natural host cells of RBSDV, GFP-fused ZmAKINβγ proteins (ZmAKINβγ-1-GFP and *ZmAKINβγ-2*-GFP) were expressed in maize protoplasts, and the GFP fluorescence was observed at 16 hpt. As shown in Figure 3A, GFP fluorescence could be observed in the nucleus and cytoplasm in ZmAKINβγ-1-GFP or *ZmAKINβγ-2*-GFP transfected protoplasts, which was the same as the fluorescence signal exhibited in free GFP-expressed control cells. Meanwhile, GFP-P8 was expressed in maize protoplasts, and the result showed that P8 could localize to the cytoplasm and nucleus, and fluorescence aggregation was found in the nucleus (Figure 3A). To evaluate the subcellular co-localization of P8 and ZmAKINβγ, GFP-P8, and RFP-fused ZmAKINβγ (ZmAKINβγ-1-RFP and *ZmAKINβγ-2*-RFP) were co-expressed in *N. benthamiana*, because of the weak fluorescence signal of RFP-fused ZmAKINβγ when co-expressed with P8 in maize protoplasts. The same as the localization observed in BiFC assay, P8 could co-localize with ZmAKINβγ-1 or -2 to the cytoplasm and nucleus in *N. benthamiana* leaf cells (Figure 3B). Furthermore, in nucleus co-expressed with P8 and free RFP, green fluorescence aggregation was found in nucleolus, in which RFP signal was not observed. In contrast, co-expression of P8 with *ZmAKINβγ-1* or *-2* disrupted the aggregation of P8 in nucleolus, suggesting the re-localization of P8 in nucleus of ZmAKINβγs-expressing *N. benthamiana* cells (Figure 3C).

### 3.4. Silencing of ZmAKINβγs Affects Primary Carbohydrate Metabolism of Maize

**Figure 4:**
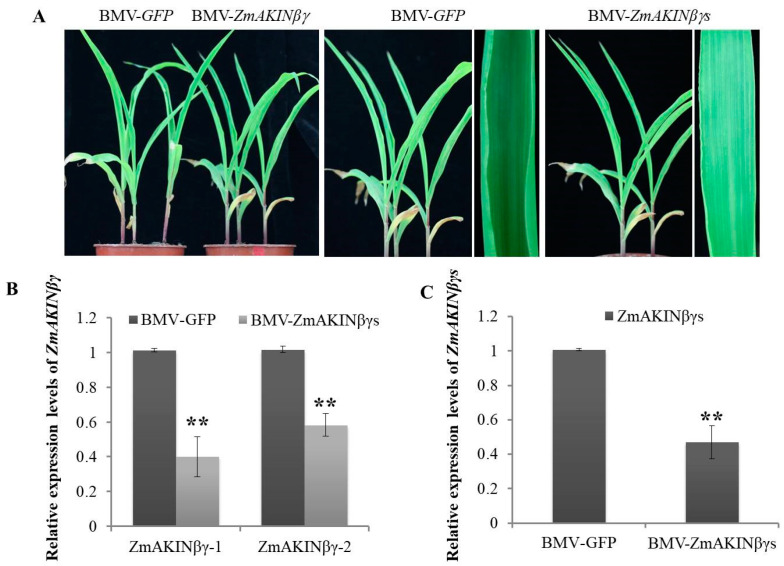
Effective knockdown of the expression of *ZmAKINβγ*s. (**A**) The phenotype of Brome mosaic virus (BMV)-GFP- or BMV-ZmAKINβγs-inoculated maize plants at 14 dpi. (**B**) Knockdown efficiency of **ZmAKINβγ-1** and *-2* in the second systemically infected leaves at 14 dpi. RT-qPCR was performed using the primer pairs specifically against each *ZmAKINβγ,* respectively. (**C**) Knockdown efficiency of *ZmAKINβγ*s in the second systemically infected leaves at 21 dpi. The primer pair located at the conserved region of two *ZmAKINβγ* genes. Three independent experiments were conducted with at least three biological replicates for each treatment. Error bars represented the means ± SE. Significant differences were indicated using Student’s *t*-test: ** indicates *p* < 0.01. Maize *ubiquitin* gene (*ZmUBI*) was used as the internal reference.

SNF1/AMPK/SnRK1 complex is a conserved core regulator of energy homeostasis in yeast, animals, and plants, which is involved in sugar sensing and dynamic regulation of metabolism [16,17,43]. In order to investigate the role of *ZmAKINβγ*s in sugar metabolism in planta, brome mosaic virus (BMV)-mediated VIGS system was recruited to downregulate the transcription levels of *ZmAKINβγ*s using previously reported methods [30,31,35]. Two *ZmAKINβγ* genes share more than 94% nucleotide identity in the coding sequences, and also have similar biological characteristics, such as the expression in different tissues, the subcellular localization, and the response to RBSDV infection (Figure 2 and Figure 3). Therefore, a fragment of conserved nucleotide sequence of **ZmAKINβγ-1** and **ZmAKINβγ-2** was cloned into brome mosaic virus (BMV) vector to target and transiently silence both *ZmAKINβγ* genes. At 14 dpi of BMV infection, tissues from the second systemically infected leaves were collected and subjected to RT-qPCR using specific primers for **ZmAKINβγ-1** and **ZmAKINβγ-2*,* respectively, to determine the gene silencing efficiency. As shown in Figure 4B, in BMV-ZmAKINβγs-inoculated maize plants, the transcription levels of target genes were downregulated by about 60% for **ZmAKINβγ-1** and about 50% for **ZmAKINβγ-2** compared with that in BMV-GFP inoculated control plants. The *ZmAKINβγ*s-silenced maize plants did not show obvious abnormal phenotype compared with the control plants (Figure 4A). At 21 dpi, primer pairs against the conserved nucleotide sequence of *ZmAKINβγ-1* and -2 were used to detect the relative expression levels of two *ZmAKINβγ* genes simultaneously, and the result showed that significant downregulation, about 50%, was retained in BMV-ZmAKINβγs-inoculated maize plants (Figure 4C).

**Figure 5:**
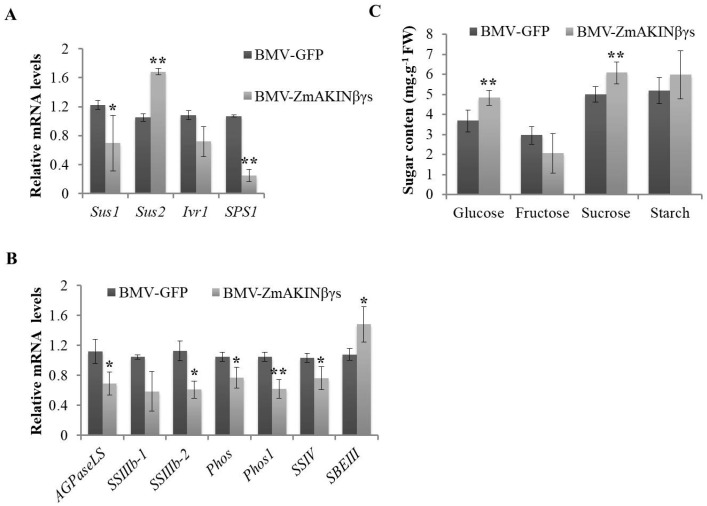
Knockdown of *ZmAKINβγ* genes regulates sugar metabolism in maize plants. (**A**) Relative expression levels of genes involved in transition between sucrose and monosaccharide. (**B**) Relative expression levels of genes involved in starch metabolism. Maize *ubiquitin* gene (*ZmUBI*) was used as the internal reference. Sus: Sucrose synthase, SPS1: Sucrose-phosphate synthase 1, Ivr1: Invertase 1, AGPaseLS: ADP-glucose pyrophosphorylase large subunit, SSIIIb: Starch synthase IIIb, SSⅣ: Starch synthase Ⅳ, Phos: Phosphoglucomutase. (**C**) The content of glucose, fructose, sucrose, and starch in BMV-ZmAKINβγs- and BMV-GFP-inoculated maize leaves. Three independent experiments were conducted with at least three biological replicates per treatment. Error bars represented the means ± SE. Significant differences were indicated using Student’s *t*-test: * indicates *p* < 0.05, ** indicates *p* < 0.01.

To further examine the effects of *ZmAKINβγ*s silencing on sugar metabolism of maize, the second systemically infected leaves were collected at 14 dpi and the transcription levels of some genes involved in sugar metabolism were analyzed by RT-qPCR. In our results, for the sucrose synthase (Sus), a critical enzyme to transform sucrose to uridine diphosphate-glucose (UDPG) and fructose, the expression of *Sus1* was downregulated, while the expression of *Sus2* was significantly upregulated (Figure 5A). The expression of a sucrose synthesis-related gene, *sucrose-phosphate synthase 1* (*SPS1*), was decreased (Figure 5A). In the starch metabolism pathway, the expression of genes involved in starch synthesis including *ADP-glucose pyrophosphorylase large subunit* (*AGPaseLS*), *starch synthase IIIb-2* (*SSIIIb-2*), *starch synthase* *Ⅳ* (*SSⅣ*), *phosphoglucomutase* (*Phos*), and *Phos1* were downregulated. In contrast, the transcription level of a gene participated in starch degradation, *starch branching enzyme III* (*SBE III*), was significantly increased (Figure 5B). Sugar metabolism is a complex physiological process in which different types of sugar or intermediate products transform reversibly in organism. To determine whether or not the expression level changes of these genes involved in several transformation pathway of sugar metabolism could eventually affect the accumulation of sugars, we measured the contents of two types of monosaccharides, glucose and fructose, and one type of oligosaccharide and polysaccharide, sucrose and starch, respectively, using the methods as previously reported [40,41,42]. In our results, the contents of glucose and sucrose in the second systemically infected leaves of *ZmAKINβγ*s-silenced plants were elevated, whereas the fructose and starch contents did not show significant changes (Figure 5C). Taken together, these results, combined with previous reports that *ZmAKINβγ*s could functionally complement yeast *snf4* (the homologue of *ZmAKINβγ*) deficiency, suggest that ZmAKINβγs could regulate sugar metabolism in maize, through functioning as a canonical γ subunit of SnRK1 complex.

### 3.5. Knockdown of ZmAKINβγs Expressions Facilitated RBSDV Accumulation in Maize

**Figure 6:**
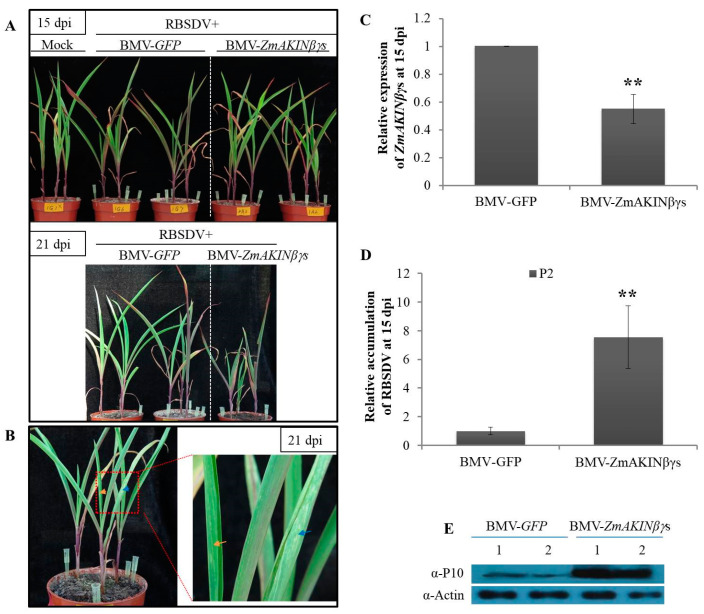
Downregulation of *ZmAKINβγ* genes facilitated RBSDV accumulation. (**A**) Phenotypes of RBSDV challenge inoculation on BMV-GFP-and BMV-ZmAKINβγs-inoculatd maize (Va35) plants. Photos were taken at 15 and 21 d post inoculation with RBSDV. (**B**) Symptoms on systemically infected maize leaves induced by BMV (indicated by blue arrow) and RBSDV (indicated by orange arrow) infections at 21 dpi. (**C**) The knockdown efficiency of *ZmAKINβγ* expressions and (**D**) relative RBSDV RNA accumulation at 15 d post inoculation (dpi) with RBSDV in the third true leaves. The quantifications of RBSDV and *ZmAKINβγ*s RNA were determined by RT-qPCR targeting P2 gene and normalized to the *UBI* served as an internal control. Three independent experiments were conducted with at least three biological replicates for each. All data are mean values±SD. Significant differences were indicated using Student’s *t*-test: ** indicates *p* < 0.01. (**E**) Accumulation levels of RBSDV P10 in the third true leaves of RBSDV challenge inoculated maize plants at 15 dpi were determined by Western blotting. The protein accumulation of Actin was used as a loading control.

In order to determine the roles of ZmAKINβγs during RBSDV infection, BMV-mediated gene silencing coupled with *L. striatellus*-mediated RBSDV inoculation was performed. As presented above, BMV-ZmAKINβγs and the control BMV-GFP were rub-inoculated to maize seedlings at two- leaf stage, respectively. Five days later, mild mosaic symptom began to emerge on the first systemically infected leaves of BMV-infected plants. Subsequently, these plants were put into a mesh enclosure, and then viruliferous (RBSDV-infected) insects of *L. striatellus* were used to inoculate RBSDV as described in previous reports [32,33]. After three days after inoculation, all insects (*L. striatellus*) were removed and plants were transferred to a chamber. As the results presented above, *ZmAKINβγ*s could be efficiently silenced by BMV VIGS system for at least 21 days (Figure 4C). In consideration of the duration of efficient target gene silencing, at 15 dpi of RBSDV, leaf tissues were collected from the third true leaves of BMV-ZmAKINβγs- or BMV-GFP-inoculated plants and subjected to further analyses, although the obvious viral symptom induced by RBSDV infection and the phenotype difference between *ZmAKINβγ*s-silenced and control plants could not yet be observed (Figure 6A). RT-qPCR analyses showed that the expression of *ZmAKINβγ*s was significantly downregulated by about 40% in BMV-ZmAKINβγs-inoculated plants compared with in control, although it has been shown in this work that RBSDV infection could elevate the transcription levels of two *ZmAKINβγ* genes (Figure 6C). Furthermore, our results showed an approximately 7-fold upregulation in RBSDV RNA accumulation and also a significant increase in RBSDV P10 protein accumulation in *ZmAKINβγ*s-silenced plants compared with BMV-GFP-inoculated control plants (Figure 6D,E). At 21 dpi, RBSDV induced swelling on the abaxial surface of maize leaves and BMV infection induced chlorotic phenotype could be observed (Figure 6B), and more severe dwarfing phenotype emerged for *ZmAKINβγ*s-silenced maize plants inoculated with RBSDV (Figure 6A). Taken together, these data indicated that simultaneous downregulation of two *ZmAKINβγ* genes could facilitate RBSDV accumulation.

## 4. Discussion

Maize rough dwarf disease caused by RBSDV is a serious threat to maize production in China. In the past several decades, a series of progresses has been made to reveal the viral characteristics or the interaction between RBSDV and plant or insect hosts. However, our understanding on the host factors responsible for RBSDV infection or plant defense is still limited. A recent report described that viral P7-1 recruited a host factor named Rab GDP dissociation inhibitor alpha (RabGDIα) for RBSDV infection in maize [2]. In this research, two copies of *ZmAKINβγ*, the regulatory subunit of SnRK1 complex were identified to interact with the P8 protein of RBSDV, and could respond to RBSDV infection in a similar response pattern. Importantly, downregulation of *ZmAKINβγ*s could facilitate RBSDV infection. Furthermore, the role of ZmAKINβγ in sugar metabolism regulation, probably through functioning as γ type subunit of SnRK1 complex, was investigated in planta.

SNF1/AMPK/SnRK1 complex, which generally consists of catalytic α and regulatory β and γ subunits in yeast and mammals, is an evolutionarily conserved core regulator for organic energy homeostasis in eukaryotic organisms [44]. In green plants, domain fused atypical βγ subunit with four CBS domains and an extended N-terminal CBM was firstly described in maize (named ZmAKINβγ) [20]. *ZmAKINβγ-1* and *ZmAKINβγ-2* could interact in vivo with ZmSnRK1a and ZmSnRK1b, two putative α subunits in maize, and could also complement the yeast *∆snf4* mutant growth deficiency on non-fermentable medium [20,29]. Furthermore, we presented in this work that knockdown of *ZmAKINβγ*s expressions influenced sugar metabolism-related gene transcriptions and the sugar contents, including glucose and sucrose, without visual effect on maize growth (Figure 5). Taken together, these results suggested that ZmAKINβγ could function as a γ subunit of SnRK1 complex in energy homeostasis. However, further investigation is needed to provide more direct evidence by using method such as native PAGE gel combined with mass spectrometry to reveal the exact composition of the functional SnRK1 complex in maize.

We demonstrated that RBSDV P8 could interact with *ZmAKINβγ-1* and *ZmAKINβγ-2* in vitro and in vivo (Figure 1). For *Arabidopsis*, previous report showed that the pre-CBS and four CBS domains (FCD) of AKINβγ are required for its γ subunit function and for the interaction with β subunit [22]. Interestingly, we found that the N terminal 237 amino acids, including the CBM, pre-CBS, and the first CBS domain of *ZmAKINβγ-2*, are indispensable for its interaction with P8 (Figure 1). Thus, it will be interesting to know whether P8 could competitively associate with ZmAKINβγ to inhibit SnRK1 complex formation and consequently interferes with its function in SnRK1 signaling pathway. On the other hand, it has been shown that ZmAKINβγ could form homodimer through the CBM or γ domain [29]. It should be further investigated whether the interaction between P8 and ZmAKINβγ could also affect any function of self-associated ZmAKINβγ.

In the past decade, significant progress has been made in understanding signal transduction mechanism underlying plant innate immunity and phytohormone action, however only limited attention was given to the interplay between plant primary metabolism and biotic defense [45]. A fine regulation of the energy metabolism, including energy sensing, intake, and expenditure is not only responsible for cellular energy provided for plant development and effective plant defense, the metabolism products, but can also be partly involved in defense signaling transduction [46,47,48]. As a global regulator of cellular energy metabolism, the function of SnRK1 complex in plant defense has been investigated in distinct plant pathosystems [45]. For example, host SnRK1α catalytic subunit was found to phosphorylate viral proteins and consequently limit geminivirus infections [26,27]. At the layer of gene expression, silencing of SnRK1α in *N. benthamiana* could enhance host susceptibility to geminivirus infection [24]. Recently, a research revealed the positive regulatory role of OsSnRK1a in rice defense against bacterial or fungal pathogens by artificially regulating the expression of this gene [49]. For the regulatory subunit of SnRK1 complex, it has been reported that *Arabidopsis* AKINβγ could interact with two proteins related to plant pathogen resistance, AtHSPRO1 and 2, suggesting its function in plant defense by direct association with resistance proteins [21]. In this work, downregulation of ZmAKINβγ genes in maize facilitated RBSDV infection, providing the first in planta evidence, to our knowledge, for the involvement of AKINβγ subunit in plant defense against viral infections (Figure 6). There are two possible reasons for this result: (1) Silencing the AKINβγ genes could limit the formation of active heterotrimer SnRK1 complex, which was reported to participate in plant defense against distinct pathogens, including viruses; and (2) the defense activity of ZmAKINβγ functioned by associating with other cellular factors was inhibited by downregulating *ZmAKINβγ* expressions. Interestingly, when we exogenously applied a certain amount of glucose to RBSDV-infected maize (which theoretically could repress the biological activity of SnRK1), viral accumulation was also increased. To some extent, this evidence tends to support the first hypothesis presented above.

Now that AKINβγ proteins of maize have been identified to interact with RBSDV P8, the biological importance and regulation mechanism underlying the direct interaction between ZmAKINβγs and P8 needs to be further investigated. ZmAKINβγs was confirmed to play roles in sugar metabolism and regulating RBSDV accumulation. Additional experiments are needed to reveal the precise relationship between maize sugar metabolism and RBSDV infection and whether ZmAKINβγ subunits or SnRK1 complex function as a node of convergence for these two biological processes.

## 5. Conclusions

To better understand the molecular interaction of RBSDV with maize hosts, in the present study, two ZmAKINβγ proteins (*ZmAKINβγ-1* and *ZmAKINβγ-2*), the putative regulatory subunit of the energy homeostasis regulator referred to SnRK1 complex, were identified to interact with P8 in vitro and in vivo. Further, the transcriptional levels of two *ZmAKINβγ* genes were found to be differentially regulated by RBSDV at different infection stage, and BMV-induced effective gene silencing of *ZmAKINβγ*s facilitated the accumulation of RBSDV, indicating the response of *ZmAKINβγ*s to RBSDV infection. In addition, downregulation of *ZmAKINβγ*s expression regulated the sugar metabolism including the gene expressions and sugar contents. Our results suggested that ZmAKINβγ proteins were involved in regulation of primary carbohydrate metabolism in maize and could function as positive regulator of hosts’ resistance to RBSDV infection.

## Supplementary Materials

The following are available online at https://www.mdpi.com/1999-4915/12/12/1387/s1, Figure S1: Multiple amino acid sequences alignment of ZmAKINβγ and homologous in rice and N. benthamiana; Figure S2: Analyses of the host-pathogen specificity of the interaction between P8 and AKINβγ by Y2H assay; Figure S3: *ZmAKINβγ-2* specifically interacted with P8 among tested nine proteins encoded by RBSDV; Figure S4: The phenotype on the back surface of the maize leaf. (A) and the accumulation levels of RBSDV P10 in the third true leaves of Mock-or RBSDV-inoculated maize plants at 20 dpi (B); Figure S5: The expression levels of *ZmAKINβγ-1* and -2 were regulated by SCMV (A) or MCMV (B) infection; Table S1: Constructs and corresponding primers used in this study; Table S2: Primers used for RT-qPCR analyses in this study.

## Abbreviations

RBSDV	Rice black streaked dwarf virus
AKINβγ	βγ subunit of *Arabidopsis* SNF1 kinase homolog
BMV	Brome mosaic virus
SCMVMCMV	sugarcane mosaic virusmaize chlorotic mottle virus
Sus	sucrose synthase
SPS	sucrose-phosphate synthase
AGPaseLS	ADP-glucose pyrophosphorylase large subunit
SSIIIb-2	starch synthase IIIb-2
SSIV	starch synthase IV
Phos	phosphoglucomutase
SBEIII	starch branching enzyme III
GFP	green fluorescence protein
YFP	yellow fluorescence protein
