# Supplementary material for: Maize AKINβγ Proteins Interact with P8 of Rice Black Streaked Dwarf Virus and Inhibit Viral Infection
Source: Viruses. 2020 Dec 4;12(12):1387. doi: 10.3390/v12121387 (PMC7761811; doi:10.3390/v12121387)
Supplement: Supplementary file 1 [file viruses-12-01387-s001.zip › Figure legends of the supplementary figures.pdf]

Supporting Information

Figure S1 Multiple amino acid sequences alignment of ZmAKINβγ and homologous in rice and *N. benthamiana*. Orange line indicated the CBM domain, and blue line indicated the first CBS domain.

Figure S2 Analyses of the host-pathogen specificity of the interaction between P8 and AKINβγ by Y2H assay. (A) Interactions between P8 and OsAKINβγ or NbAKINβγ in yeast. (B) Interactions between ZmAKINβγ and SP8 of SRBSDV in yeast. The plasmid combinations indicated were co-transformed into yeast strain Y2H Gold, and the transformants were spotted on the selective medium SD/-Trp/-Leu and SD/-Trp/-Leu/-His/-Ade with 10-fold serial dilutions and grown at 30°C for 3-4 d.

Figure S3 ZmAKINβγ-2 specifically interacted with P8 among tested nine proteins encoded by RBSDV. The plasmid combinations indicated were co-transformed into yeast strain Y2H Gold, and the transformants were spotted on the selective medium SD/-Trp/-Leu/-His/-Ade and grown at 30°C for 3-4 d.

Figure S4 The phenotype on the back surface of the maize leaf. (A) and the accumulation levels of RBSDV P10 in the third true leaves of Mock-or RBSDV-inoculated maize plants at 20 dpi (B). The P10 accumulations were determined by western blotting. The orange arrow indicates the white swelling. CBB: Coomassie Brilliant Blue.

Figure S5 The expression levels of *ZmAKINβγ-1* and *-2* were regulated by SCMV (A) or MCMV (B) infection. The second systemic infected leaves were collected from mock- and SCMV-or MCMV-inoculated maize plants at the indicated time points and subjected for RT-qPCR. Three independent experiments were conducted with at least three biological replicates per treatment. Error bars represented the means±SE. Significant differences were indicated using Student's t-test: * indicates $P < 0.05$, ** indicates $P < 0.01$.
