# Supplementary material for: Maize AKINβγ Proteins Interact with P8 of Rice Black Streaked Dwarf Virus and Inhibit Viral Infection
Source: Viruses. 2020 Dec 4;12(12):1387. doi: 10.3390/v12121387 (PMC7761811; doi:10.3390/v12121387)
Supplement: Supplementary file 1 [file viruses-12-01387-s001.zip › Supplemental Table S1.pdf]

Supplemental Table S1. Constructs and corresponding primers used in this study

Construct	Vector	Primer sequence (5'-3')
**Y2H assay**		
BD-P8	pGBKT7	F: ATTCCATGGAGATGACTGGCACCCATGAC<br>R: GCCGGATCCCTTACACAATAATAGATGCAG
BD-SP8	pGBKT7	F: GCTCATATGATGATTGGCACCTATGATGA<br>R: ATTGGATCCCTTAGCAAAGAATAGACACAGC
BD-P5-1	pGBKT7	F: ATTCCCGGGTATGACATATTCGAAAGTG<br>R: TAAGTCGACGTCACGTTGAAGATGGTTG
BD-P5-2	pGBKT7	F: ATTCCATGGAGATGACAAAATTTCCACTTG<br>R: GAAGGATCCCTCAGAGATGAAGCATGAC
BD-P6	pGBKT7	F: ATTCCATGGAGATGTCTGCCCACCTGACC<br>R: GCCGGATCCCTTACTCAGAGCTTAGTTGCC
BD-P7-1	pGBKT7	F: ATTGGATCCGTATGGATAGACCTGCTCGA<br>R: GCCCTGCAGGTTAAGCAGAAGGAGATGA
BD-P7-2	pGBKT7	F: GATCCATGGAGATGAATTACACTTTAGG<br>R: CTCGGATCCCTTAAGAATTCAGTATCTT
BD-P9-1	pGBKT7	F: ATTCCATGGAGATGGCAGACCAAGAGC<br>R: CCAGGATCCCTCAAACGTCCAATTTC
BD-P9-2	pGBKT7	F: ATTCCATGGAGATGAATCCCCAATCTT<br>R: CACGGATCCCTTAATTAAAAAGCGTATAG
BD-P10	pGBKT7	F: ATTCCATGGAGATGGCTGACATAAGACTC<br>R: GATGGATCCCTCATCTTGTCACTTTGTT
AD-ZmAKINβγ-2	pGADT7	F: ATTGAATTCATGTTCTCGCACGGCGCTG<br>R: CACGGATCCCTCACAAGCTCAGCAAGAAC
AD-ZmAKINβγ-1	pGADT7	F: ATTGAATTCATGTTCTCGCACGGCGC<br>R: CCTGGATCCCTCACAAGCTCAGCAAGAAC
AD-OsAKINβγ	pGADT7	F: ATTATCGATACATGTTTTCCCACGGCGC<br>R: GCCCTCGAGCTTAGCTCAGCAAGAACTTG
AD-NbAKINβγ	pGADT7	F: ACGATCGATACATGTTTGGGTCTGGGAG<br>R: ATTGGATCCCGCAGCCGAGCAAGAAC
AD-ZmAKINβγ-2(1-153)	pGADT7	F: The same as for AD-ZmAKINβγ-2<br>R: GCAGGATCCTCACTCTGAAACTCTCAGAGTAC
AD-ZmAKINβγ-2(154-496)	pGADT7	F: GCCGAATTCATGGCTGCAATACAAATATCTAG<br>R: ATTGGATCCTCACAAGCTCAGCAAGAAC
AD-ZmAKINβγ-2(1-237)	pGADT7	F: The same as for AD-ZmAKINβγ-2<br>R: ACTGGATCCCCGAGCCATGAGTTTCTAG
AD-ZmAKINβγ-2(1-333)	pGADT7	F: The same as for AD-ZmAKINβγ-2<br>R: GCTGGATCCCAGTTGAGTTTTTGAAGTATC
AD-ZmAKINβγ-2(1-415)	pGADT7	F: The same as for AD-ZmAKINβγ-2<br>R: GCTGGATCCCTGTGTAGACCTTGTCTTT
**BiFC assay**		
P8-YFP^{N}	pSPYNE-35S	F: CAGACTAGTATGACTGGCACCCATGAC<br>R: GACCTCGAGCACAATAATAGATGCAGC

P8-YFP^{C}	pSPYCE-35S	F: The same as for P8-YFP^{N}<br>R: The same as for P8-YFP^{N}
ZmAKINβγ-2-YFP^{C}	pSPYCE-35S	F: ATTACTAGTATGTTCTCGCACGGCGCTG<br>R: AGACTCGAGCAAGCTCAGCAAGAACTTG
ZmAKINβγ-2-YFP^{N}	pSPYNE-35S	F: The same as for ZmAKINβγ-2-YFP^{C}<br>R: The same as for ZmAKINβγ-2-YFP^{C}
ZmAKINβγ-1-YFP^{C}	pSPYCE-35S	F: The same as for ZmAKINβγ-2-YFP^{C}<br>R: The same as for ZmAKINβγ-2-YFP^{C}
pUC-P8-YFP^{N}	pUC-SPYNE	F: The same as for P8-YFP^{N}<br>R: The same as for P8-YFP^{N}
pUC-ZmAKINβγ-2-YFP^{C}	pUC-SPYCE	F: The same as for ZmAKINβγ-2-YFP^{C}<br>R: The same as for ZmAKINβγ-2-YFP^{C}
pUC-ZmAKINβγ-1-YFP^{C}	pUC-SPYCE	F: The same as for ZmAKINβγ-2-YFP^{C}<br>R: The same as for ZmAKINβγ-2-YFP^{C}
**Subcellular localization**		
ZmAKINβγ-1-GFP	pGD-eGFP	F: ATTAGATCTATGTTCTCGCACGGCGC<br>R: CAAGTCGACCAAGCTCAGCAAGAACTTG
ZmAKINβγ-2-GFP	pGD-eGFP	F: The same as for ZmAKINβγ-1-GFP<br>R: The same as for ZmAKINβγ-1-GFP
GFP-P8	pGD-eGFP	F: ATTCTGCAGTCATGACTGGCACCCATGA<br>R: CGCGGATCCTTACACAATAATAGATGCAG
ZmAKINβγ-1-RFP	pGD-RFP	F: The same as for ZmAKINβγ-1-GFP<br>R: The same as for ZmAKINβγ-1-GFP
ZmAKINβγ-2-RFP	pGD-RFP	F: The same as for ZmAKINβγ-1-GFP<br>R: The same as for ZmAKINβγ-1-GFP
**BMV VIGS**		
BMV-*ZmAKINβγ*s	pC13/F3	F: ATTCCTAGGAACAGTCGTCCATTGGC<br>R: ACTCCATGGCGAACATGTGTGTAGAC

The single-underlined letters indicate the enzymatic restriction sites
