# Supplementary material for: Maize AKINβγ Proteins Interact with P8 of Rice Black Streaked Dwarf Virus and Inhibit Viral Infection
Source: Viruses. 2020 Dec 4;12(12):1387. doi: 10.3390/v12121387 (PMC7761811; doi:10.3390/v12121387)
Supplement: Supplementary file 1 [file viruses-12-01387-s001.zip › Supplemental Table S2.pdf]

Supplemental Table S2. Primers used for RT-qPCR analyses in this study

Primer name	Primer sequence (5'-3')	Usage
qZMAK1-F	ATGGAGCGACTGGCTAATCC	RT-qPCR analysis of *ZmAKINβγ-1* gene
qZMAK1-R	CAGACCATGAATTCCAACAGCC	
qZMAK2-F	TGAAGGTGATGGAGCGACTG	RT-qPCR analysis of *ZmAKINβγ-2* gene
qZMAK2-R	CCCTGACGATAGCCAATTATCC	
qZMAK-F	CATTGGCTATGTTGCGACCT	RT-qPCR analysis of *ZmAKINβγ* genes simultaneously
qZMAK-R	AAAGGCGTATTGGCATCTTG	
qP2-F	CACAAAGAGAGCGAGAGTGACA	RT-qPCR analysis of RBSDV
qP2-R	TGTAGCAGGTTGAACTGGGTC	
qIvr1-F	GGCAACGACGACTTCTCTATGA	RT-qPCR analysis of *invertase1* gene
qIvr1-R	TGCTCCCTGCTCCCTCTTAT	
qSuS2-F	TCTATGAAGCGTTCGGTCTCA	RT-qPCR analysis of *sucrose synthase 2* gene
qSuS2-R	GCCATCAGATTAGCAGCCTG	
qSuS1-F	GGAGAACGAAATGCTGCTGA	RT-qPCR analysis of *sucrose synthase 1*
qSuS1-R	TCCACTTGCGAACGATTCC	
qSPS1-F	GCTTCAGGTTGTCCCTCTGC	RT-qPCR analysis of *sucrose phosphate synthase 1* gene
qSPS1-F	TCCTCACCAGTGCTTCCGAA	
qPhos-F	CCCAAGGATGCCAAGGAAA	RT-qPCR analysis of *Phosphoglucomutase* gene
qPhos-R	GCACGCTTTGACGAATGTG	
qSSIV-F	GCGAAATGGCTTCACCTTT	RT-qPCR analysis of *starch synthase IV* gene
qSSIV-R	TCTGCTGTTTATGCCGCTG	
qSSIIIb2-F	CAAGAGGGTAATGGAGCAAGA	RT-qPCR analysis of *starch synthase IIIb-2* gene
qSSIIIb2-R	ACGTCGTACTGTTGTTTTGCA	
qSSIIIb1-F	GCATTGGTGTTGGATTGGG	RT-qPCR analysis of *starch synthase IIIb-1* gene
qSSIIIb1-R	CAGCCTTTATTCTTTCGGCAC	
qAGPaseLS-F	TTGTTGGAGTTCGTTCTCGG	RT-qPCR analysis of *ADP-glucose pyrophosphorylase large subunit* gene
qAGPaseLS-R	CCCTATCCTCGCATTCTTGTC	
qPhos1-F	TCTTTGTGACACCGTCGGA	RT-qPCR analysis of *Phosphoglucomutase 1* gene
qPhos1-R	TCCATCCAGTAGGCACCTCA	
qSBEIII-F1	GCTCTGTTGGTTCTGTTGACG	RT-qPCR analysis of *starch branching enzyme III* gene
qSBEIII-R1	AGTTCCCATTGCCGATTAGC	
qUBI-F	GGAAAAACCATAACCCTGGA	RT-qPCR analysis of *Ubiquitin* gene as internal control
qUBI-R	ATATGGAGAGAGGGCACCAG	
