# Supplementary figures and images for: Maize AKINβγ Proteins Interact with P8 of Rice Black Streaked Dwarf Virus and Inhibit Viral Infection
Source: Viruses. 2020 Dec 4;12(12):1387. doi: 10.3390/v12121387 (PMC7761811; doi:10.3390/v12121387)

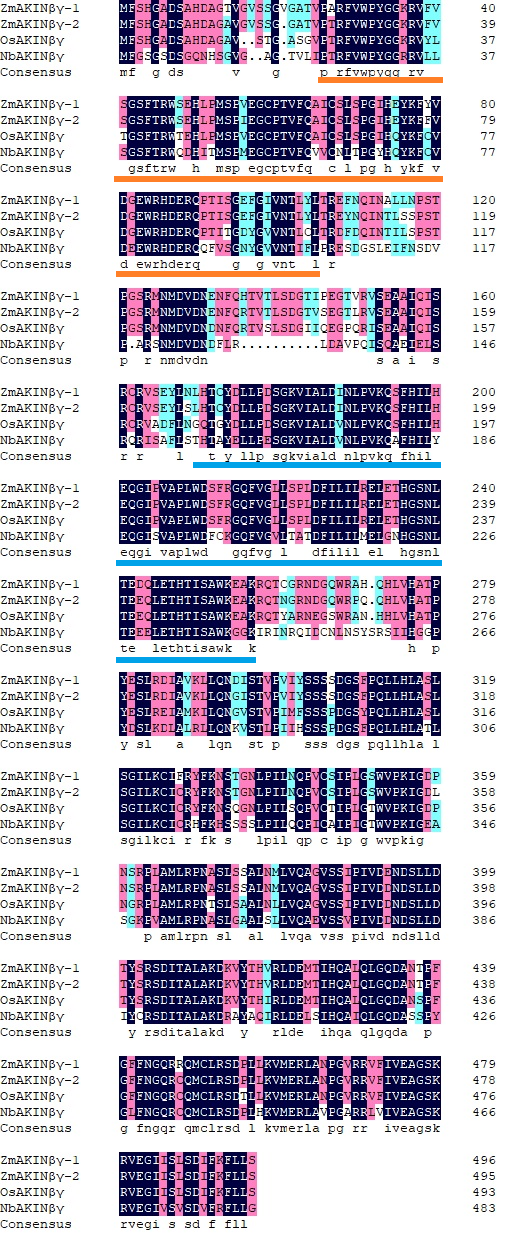

Supplement: Supplementary file 1 [file viruses-12-01387-s001.zip › Fig S1.png]

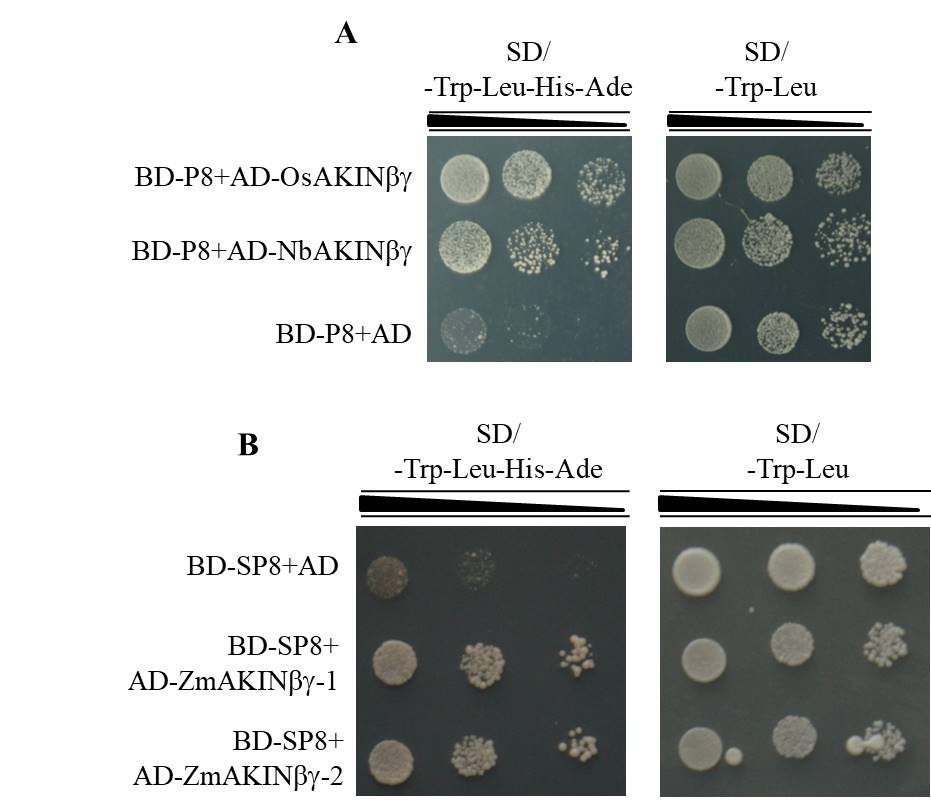

Supplement: Supplementary file 1 [file viruses-12-01387-s001.zip › Fig S2.jpg]

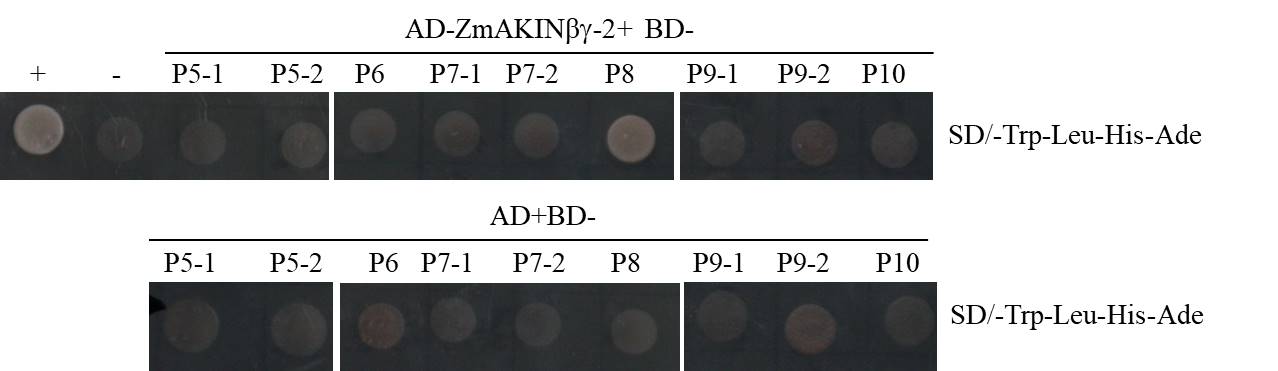

Supplement: Supplementary file 1 [file viruses-12-01387-s001.zip › Fig S3.jpg]

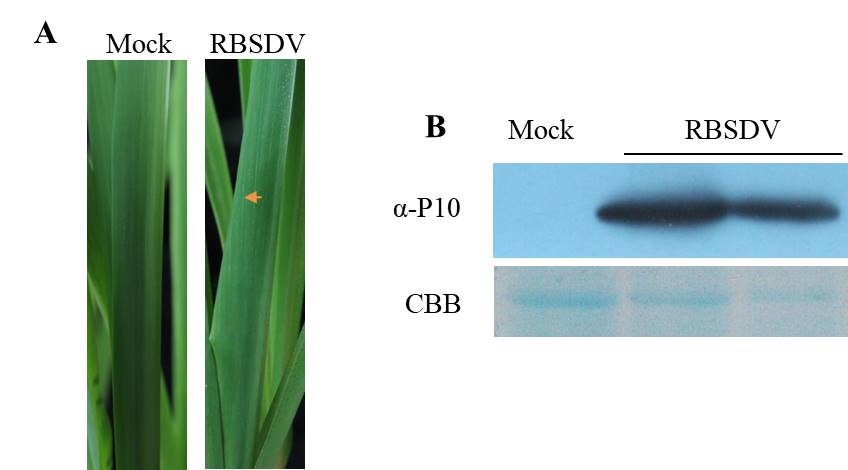

Supplement: Supplementary file 1 [file viruses-12-01387-s001.zip › Fig S4.jpg]

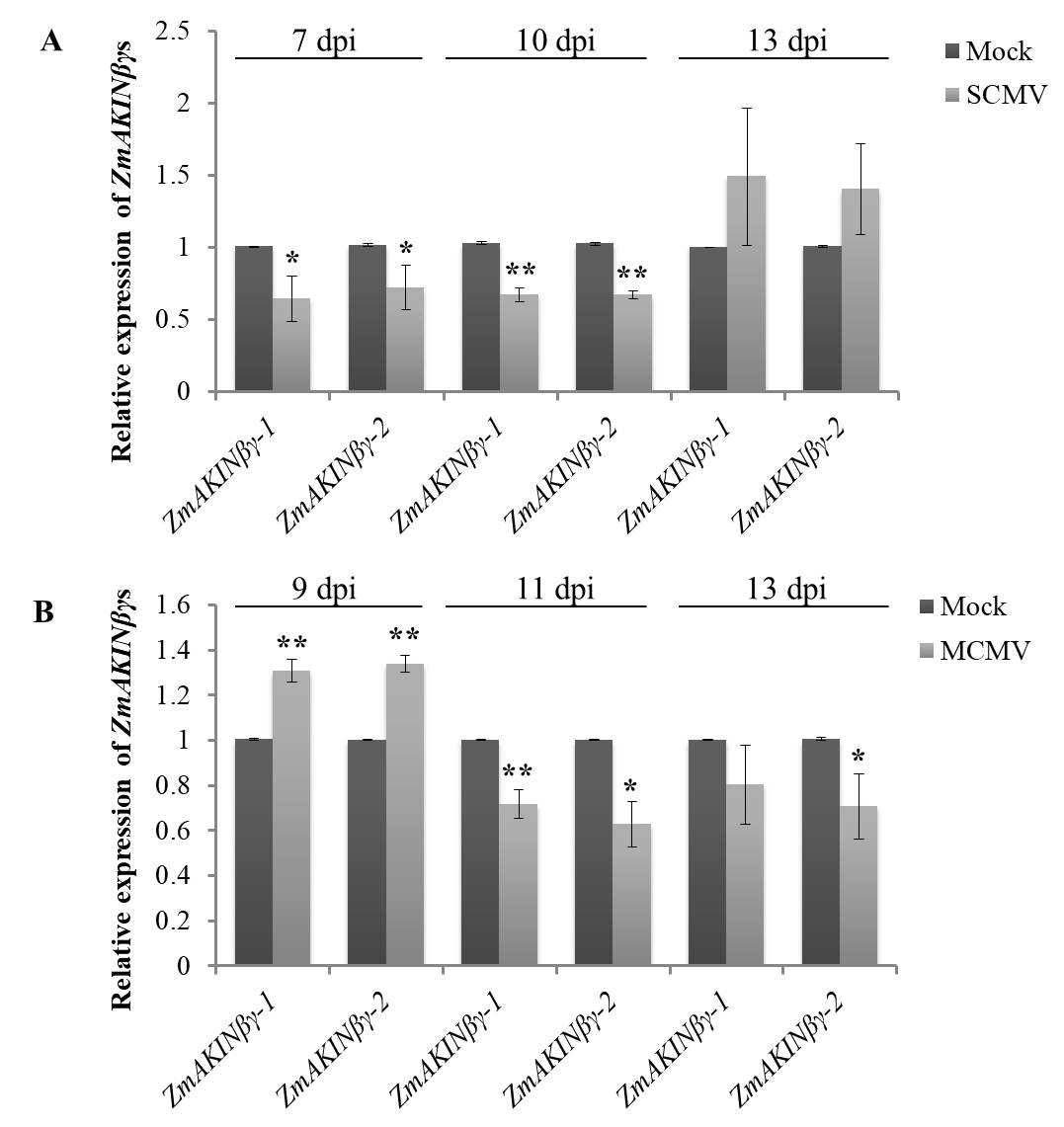

Supplement: Supplementary file 1 [file viruses-12-01387-s001.zip › Fig S5.jpg]
